# Infection and transmission dynamics of rKSHV.219 in primary endothelial cells

**DOI:** 10.1016/j.jviromet.2013.06.001

**Published:** 2013-10

**Authors:** Hannah C. Jeffery, Rachel L. Wheat, David J. Blackbourn, Gerard B. Nash, Lynn M. Butler

**Affiliations:** a School of Cancer Sciences, College of Medical and Dental Sciences, University of Birmingham, B15 2TT, UK; b School of Clinical and Experimental Medicine, College of Medical and Dental Sciences, University of Birmingham, B15 2TT, UK

**Keywords:** Endothelial cell, Kaposi's sarcoma-associated herpes virus, Rkshv.219

## Abstract

Kaposi's sarcoma-associated herpesvirus (KSHV) is the aetiologic agent of Kaposi's sarcoma (KS), a tumour of endothelial cell origin. The study of KS development was aided by the generation of a recombinant GFP (latent)/RFP (lytic)-expressing KSHV (rKSHV.219) by Vieira and O’Hearn (2004). In this study the first data characterising primary endothelial cell infection and transmission with this virus is presented. Infection was predominantly latent and the percentage of GFP-positive cells increased over time. Neither horizontal transmission of infection, nor cellular proliferation, explained this increase. Analysis of latency-associated nuclear antigen (LANA-1) expression revealed that a threshold level of infection was required for GFP expression early post infection. At later time points GFP correlated more closely with LANA-1 expression, likely due to the accumulation of GFP over time. This study provides methodological guidance for the use of rKSHV.21. In addition, it highlights potential problems associated with the use of fluorescent proteins as markers of viral infection.

## Highlights

• We examined infection and transmission of rKSHV.219 in endothelial cell cultures.
• The number of rKSHV.219 infected/GFP positive endothelial cells increased over time.
• This increase was not due to horizontal transmission or cellular proliferation.
• LANA analysis revealed an infection threshold was required for GFP expression.
• GFP was a reliable indicator of infection only at later time points post infection.

## 1 Introduction

Kaposi's sarcoma-associated herpesvirus (KSHV) is the aetiologic agent of Kaposi's sarcoma (KS), a multifocal malignancy characterised by spindle-shaped tumour cells with an endothelial phenotype (Ganem, 2006). KS is currently the leading malignancy associated with acquired immunodeficiency syndrome (AIDS) and endemic KS is a cause of high morbidity and mortality in parts of Africa (Mesri et al., 2010). Historically, most *in vitro* studies of the consequences of KSHV infection of endothelial cells have used concentrated virus from supernatants of primary effusion lymphoma cultures, which had been induced into lytic replication using phorbyl myristate acetate. Although cell lines can be readily infected with KSHV produced in this way, primary endothelial cells are less susceptible, with some reporting very low (<10%) KSHV infection rates using standard protocols (Ciufo et al., 2001, Flore et al., 1998). Others achieved higher infection rates with the aid of the antiheparin reagent, polybrene (DiMaio et al., 2011), but at the expense of possible off target effects. Thus, it is important to be able to identify KSHV-infected endothelial cells from uninfected endothelial cells within the inoculated population, particularly when infection rates are low. However, endothelial cells infected with primary effusion lymphoma cell-derived KSHV cannot be readily distinguished from uninfected endothelial cells without staining for KSHV antigens (such as the nuclear expressed latency-associated nuclear antigen, LANA-1). To circumvent this inconvenience, and to also allow a platform for genetic manipulation of KSHV, Vieira and O’Hearn generated a novel recombinant KSHV (rKSHV.219), propagated in the primate Vero cell line. This virus was constructed using KSHV from the JSC-1 primary effusion lymphoma cell line and was engineered to expresses the green fluorescent protein (GFP) gene from the EF-1α promoter, as a marker of latent infection, and the red fluorescent protein (RFP) gene from the PAN RNA promoter, as a lytic cycle marker (Vieira and O’Hearn, 2004). The generation of this recombinant virus made the identification of ‘rKSHV.219-infected’ cells (GFP-positive) and ‘rKSHV.219 lytic’ cells (RFP-positive) very convenient. For these reasons many groups, including our own, have used rKSHV.219 to study the consequences of KSHV-infection on endothelial cells and other cell types.

This study describes the infection dynamics of rKSHV.219 in primary endothelial cells (isolated from human umbilical veins) and evaluates the validity of using GFP as a definitive marker of infection. In the system, the peak in RFP-positive, lytic cells occurred early after inoculation and the percentage of GFP-positive cells in rKSHV.219-inoculated cultures increased over time. Importantly, this increase in GFP-positive cells was not due to the induction of infected cell proliferation. Neither was it caused by transmission of the virus from the lytically infected to the uninfected cells within the population. Instead, the observations in this study suggested that the temporal increase in percentage GFP-positive cells within inoculated cultures was due to the accumulation of cellular GFP over time, rather than de novo infection. Moreover, this study identified that at early time points post-inoculation GFP-negative endothelial cells could be positive for LANA-1; thus it highlighted a discrepancy between the two alternative systems for detection of infection that this model provides (percentage GFP-positivity and positivity for a KSHV latency protein such as LANA-1). GFP-negative, LANA-1 positive endothelial cells had a lower number of LANA-1 dots than those that were GFP-positive, suggesting that a threshold level of infection was necessary for GFP expression to reach detectable levels. Greater concordance between LANA-1 and GFP expression was noticed at later times post-inoculation, indicating that GFP became a more reliable marker of infection over time.

Overall, this report provides important guidance for the use of rKSHV.219 in studies of primary endothelial cell infection with KSHV. In addition to their importance in the context of the interpretation of experimental results acquired using rKSHV.219, these observations highlight potential problems when using GFP expressed from a cellular promoter as a definitive marker of viral infection at early time points. In addition, this study highlights issues that should also be considered in the context of other recombinant viruses that have been similarly engineered to express fluorescent proteins as markers of infection. Furthermore, it reveals the heterogeneity of primary endothelial cells for infection with rKSHV.129 and provides novel insights into the biology of KSHV cellular dissemination within primary endothelial cell cultures.

## 2 Materials and methods

### 2.1 Production of rKSHV.219 from VK219 cells

rKSHV.219 was produced from the latently infected Vero cell line, VK219. VK219 cells were maintained at 37 °C, 5% CO_2_ in MEM medium (Sigma, Poole, UK) supplemented with 10% foetal bovine serum (FBS; PAA Laboratories, Yeovil, UK), 2.2 g/L NaHCO_3_, 5 μg/ml puromycin (both Sigma), 10 U/ml penicillin and 10 μg/ml streptomycin (both Invitrogen, Life Technologies, Carlsbad, CA). For rKSHV.219 production, VK219 were plated to yield 60% confluence and infected with Baculovirus K50 (BacK50) with 1.25 mM sodium butyrate (Sigma), to induce lytic replication. BacK50 is a recombinant baculovirus engineered to express the KSHV lytic switch replication and transcription activator (RTA) protein, encoded by ORF50 (Vieira and O’Hearn, 2004). BacK50 was produced in insect SF9 cells maintained at 28 °C, 5% CO_2_ in Sf-900 II medium (Invitrogen) supplemented with 10% FBS (PAA Laboratories), 50 U/ml penicillin and 50 μg/ml streptomycin (both Invitrogen). For BacK50 production, SF9 cells were expanded to 2 × 10^6^ cells/ml and inoculated with BacK50. At 3 days post-inoculation (dpi), supernatants were separated from SF9 cells by centrifugation at 450 × *g* for 20 min, filtered through a 0.45 μm filter and stored at 4 °C. 48 h following BacK50-induced reactivation, VK219 cell supernatants were collected and centrifuged at 500 × *g* for 15 min, to remove cell debris, then ultracentrifuged at 31,000 × *g*, 4 °C for 4 h. The resultant rKSHV.219 pellet was re-suspended overnight in EBM2 medium (Lonza, Clonetics, Anaheim, CA, USA). We tested the effect of storage temperature (4 °C or −80 °C) on rKSHV.219 infectious titre. One week post-production there was no effect of storage temperature on infectious titre. However, after 3–4 weeks we noticed that storage at −80 °C tended to reduce infectious titre compared to storage at 4 °C (supplementary Figure 1). Therefore, rKSHV.219 preparations were subsequently stored at 4 °C prior to use.

### 2.2 Determination of rKSHV.219 titre

Approximate infectious titre of rKSHV.219 was determined using a standardised protocol of titration on HEK293 cells. HEK293 cells were maintained at 37 °C, 5% CO_2_ in Dulbecco's Modified Eagle Medium (Gibco, Invitrogen) supplemented with 10% FBS (PAA Laboratories), 2 mM glutamine, 50 U/ml penicillin, 50 μg/ml streptomycin (all Invitrogen) and 1% non-essential amino acids (Sigma). HEK293 were plated at 2.0 × 10^4^ cells/96-well then cultured overnight before serial dilutions of rKSHV.219, diluted in EBM2 medium, were added and the plates centrifuged at 330 × *g* for 30 min. The medium was changed after 90 min and the number of GFP-positive cells per well was quantified 48 h later. The titre was calculated (infectious units/ml), assuming that one infectious unit generated one GFP-positive HEK293. We found a reduction in rKSHV.219 infectious titre over extended storage time (supplementary Figure 2). Therefore, the infectious titres of preparations were re-determined if they were not used within 3 weeks of production.

### 2.3 Isolation and culture of human umbilical vein endothelial cells

Primary endothelial cells were isolated from umbilical cords and cultured in Medium 199 (Gibco, Invitrogen) supplemented with 20% FBS, 2.5 μg/ml amphotericin B, 50 U/ml penicillin, 50 μg/ml streptomycin, 1 ng/ml EGF, 28 μg/ml gentamycin and 1 μg/ml hydrocortisone (all Sigma). Primary cultures were dissociated with trypsin/EDTA (Sigma) and passaged into tissue culture multi-well plates (Falcon; Becton Dickinson Labware, Oxford, UK) or pre-fabricated channel slides (μ-Slide VI) (Ibidi, Martinsried, Germany). Seeding density yielded confluent monolayers for infection within 24 h, unless otherwise stated. Each experiment used a separate endothelial cell donor.

### 2.4 Infection of endothelial cells with rKSHV.219

rKSHV.219 was diluted in EBM2 (Lonza, Clonetics) to give appropriate infectious units/ml prior to addition to endothelial cells at a multiplicity of infection (MOI) of between 1 and 10. Inoculated cells were centrifuged at 450 × *g* for 30 min (untreated samples were treated in an identical manner, except there was no addition of virus). Endothelial cells were incubated for a further 90 min at 37 °C, 5% CO_2_ before inoculum was replaced with M199 and supplements. rKSHV.219-inoculated and untreated endothelial cells are denoted as rKSHV.219-EC and UT-EC, respectively in the figures.

### 2.5 Determining the percentage of GFP-positive, rKSHV.219-infected cells within inoculated endothelial cell cultures

Cultures of untreated and KSHV.219-inoculated endothelial cells were dissociated with trypsin-EDTA (Sigma) and fixed in 2% formaldehyde (Sigma). The GFP fluorescence intensity of each culture was analysed on a Dako Cyan flow cytometer. A minimum of 3000 events was counted. Data were analysed using FlowJo (Tree Star, OR, USA) or Dako Summit 4.3 (Dako, Stockport, UK) software. Cells were considered GFP-positive if they had a GFP fluorescence intensity greater than that of 99% of untreated cells.

### 2.6 Immunocytochemistry for LANA-1 expression

Endothelial cells were fixed in 4% formaldehyde, permeabilised with methanol and blocked with PBS/goat serum (5%) (Sigma). Primary antibody against LANA-1 (unconjugated rat anti-LANA, Advanced Biotechnologies, Columbia, USA) was added and incubated overnight at 4 °C then conjugated secondary antibody (Alexa Fluor 633-conjugated anti-rat IgG, Invitrogen) was added for 1–2 h. Bisbenzimide (Sigma) was used as a nuclear stain and channels were coated with ProLong Gold anti-fade mountant (Invitrogen) prior to visualisation. Images were captured on a Zeiss LSM510 confocal microscope and analysed using ImageJ software. For each cell, the number of LANA-1 dots and its GFP status was recorded.

### 2.7 Assay for the effect of rKSHV.219-inoculation upon endothelial cell density over time

Endothelial cells were plated in pre-fabricated channel slides (μ-slides VI; Ibidi), cultured overnight and then inoculated with rKSHV.219 at MOI10. At time points up to 10 dpi, cultures were observed using a 4× objective lens and phase contrast, GFP fluorescence (signifying latent infection) and RFP fluorescence (signifying lytic infection) images were taken. After imaging, monolayers were washed four times with complete medium and images retaken so that the number of lytic cells not removed by washing could be subtracted from the number observed at the next time point and the extent of lytic reactivation between time points calculated. The cumulative evolution of lytic cells was also calculated by summing the numbers generated between time points. The viable cell density was determined from the phase contrast images.

### 2.8 Assay to examine horizontal transmission of infection through rKSHV.219-inoculated endothelial cell monolayers

To determine if rKSHV.219 was transmitted horizontally through endothelial cell monolayers, rKSHV.219-inoculated cells were co-cultured with untreated cells that had been labelled with the far-red fluorescent tracer DDAO-SE (Invitrogen). rKSHV.219 transmission to untreated cells could be identified by dual fluorescence for GFP and DDAO-SE. For the assay, three populations of cells (rKSHV.219-inoculated non-labelled; untreated non-labelled and untreated DDAO-SE labelled) were detached from the inoculation plate at the time point of interest, either 5 h or 3 dpi, and re-plated in a 48-well plate either as mono-cultures or as 1:1 co-cultures including: (i) untreated DDAO-SE labelled + untreated non-labelled and (ii) untreated DDAO-SE labelled + rKSHV.219-inoculated non-labelled. Aliquots of each cell suspension were transferred to FACS tubes at the start of the co-culture period and fixed in 2% formaldehyde for assessment of the starting/background percentages of each cell type which included: rKSHV.219 inoculated but non-infected (DDAO-SE negative/GFP negative); rKSHV.219 inoculated infected (DDAO-SE negative/GFP-positive); untreated uninfected (DDAO-SE positive/GFP negative); untreated infected (DDAO-SE positive/GFP-positive). After 5 days of co-culture, cells were detached and fixed in 2% formaldehyde (Sigma) for analysis of the extent of transmission by flow cytometry.

### 2.9 Statistics

Parametric statistical tests were performed using GraphPad Prism 5.0a following testing to confirm that data were normally distributed using the Kolmogorov–Smirnov test. Unless otherwise stated in the figure legends, effects of treatments were tested using analysis of variance (ANOVA) followed by the Dunnett's multiple comparison or Bonferroni post hoc tests. Unpaired *t*-tests or paired *t*-tests as appropriate were used to compare characteristics of cells found in two different populations or at two different time points. Error bars on graphs represent the standard error of the mean (SEM).

## 3 Results

### 3.1 Infection of endothelial cell cultures with rKSHV.219

**Fig. 1:**
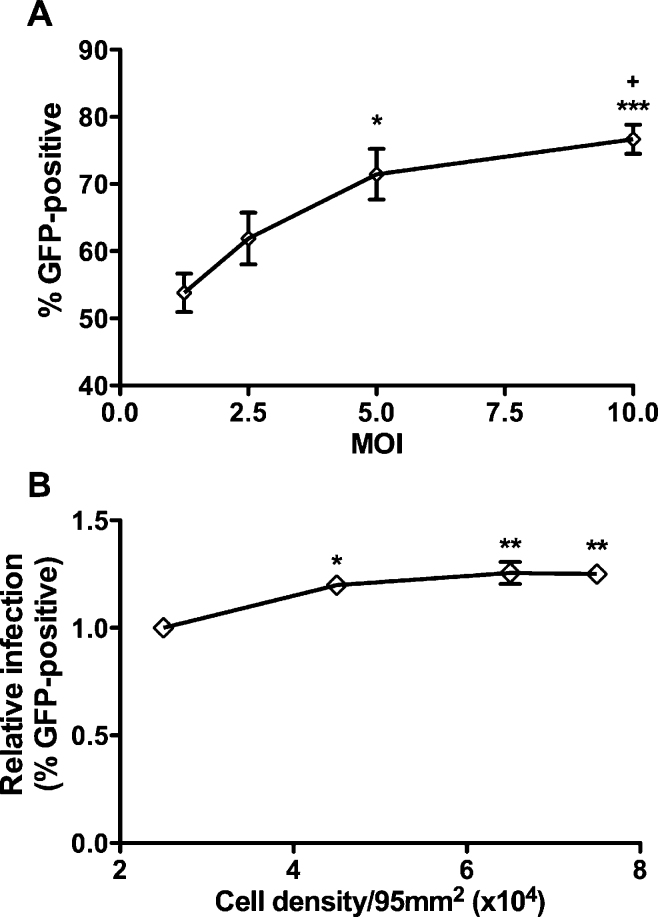
Effect of rKSHV.219 MOI and confluence on GFP expression. (A) Endothelial cells were inoculated with rKSHV.219 at multiplicities of infection (MOI) between 1.25 and 10 and cultured for a further 10 days, before the percentages of GFP-positive cells were measured by flow cytometry (*P < 0.05; ***P < 0.001 compared to MOI1.25 and ^+^*P* < 0.05 compared to MOI 2.5). (B) Endothelial cells were plated at densities between 2.5 and 7.5 × 10^4^ cells/95 mm^2^, inoculated with rKSHV.219 (MOI10) and cultured for a further 7 days, before the percentages of GFP-positive cells were measured by flow cytometry. Data are expressed relative to the lowest density tested (*P < 0.05; **P < 0.01 compared to a density of 2.5 × 10^4^ cells/95 mm^2^). Data are mean ± SEM for 3–6 independent experiments.

In order to determine the effect of virus copy number on the percentage of GFP-positive cells within cultures of rKSHV.219-inoculated endothelial cells, rKSHV.219 was added at different multiplicities of infection (MOI), and the percentage of GFP-positive cells determined at 10 dpi using flow cytometry. The number of GFP-positive cells increased with MOI (Fig. 1A); therefore, a MOI of 10 was used in all subsequent experiments to ensure maximal infection. Previous studies have often published methods inoculating sub-, rather than fully confluent, cell monolayers with KSHV, presumably due to the widely held assumption that infection of cells with viruses is more efficient under these conditions (Billstrom et al., 1999, Ciufo et al., 2001, DeMarchi and Kaplan, 1977, Moses et al., 1999). In order to investigate if this was true for rKSHV.219 infection of endothelial cells, monolayers of varying cell densities between sub- and fully confluent were treated with the virus and the percentage of GFP-positive cells assessed at 7 dpi. Interestingly, infection was least efficient in the most sparsely seeded monolayer, and there was no difference in the percentage of GFP-positive cells between the other densities tested (Fig. 1B). All subsequent experiments were performed using a cell number that gave a fully confluent monolayer at the time of inoculation.

### 3.2 Characterisation of rKSHV.219 GFP and RFP expression in endothelial cells

**Fig. 2:**
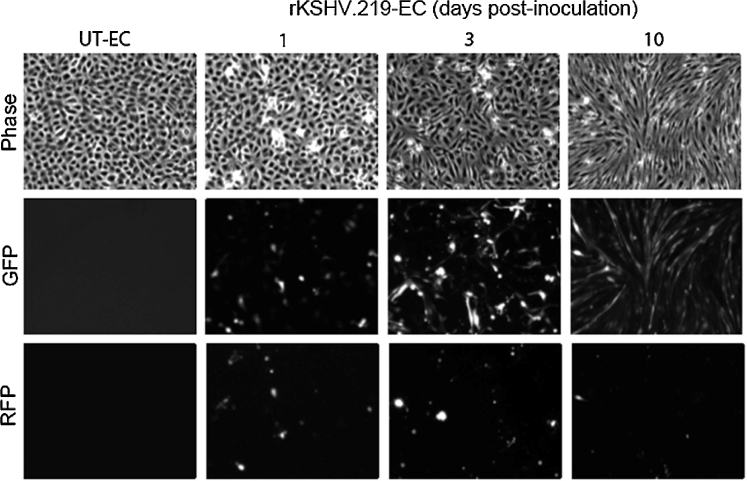
Effect of time post rKSHV.219-inoculation on cell morphology, GFP expression and RFP expression. Endothelial cells were untreated or inoculated with rKSHV.219 (MOI 10). Phase contrast, GFP and RFP fluorescence images were taken up to 10 dpi. The panel shows representative images of 3 independent experiments.

**Fig. 3:**
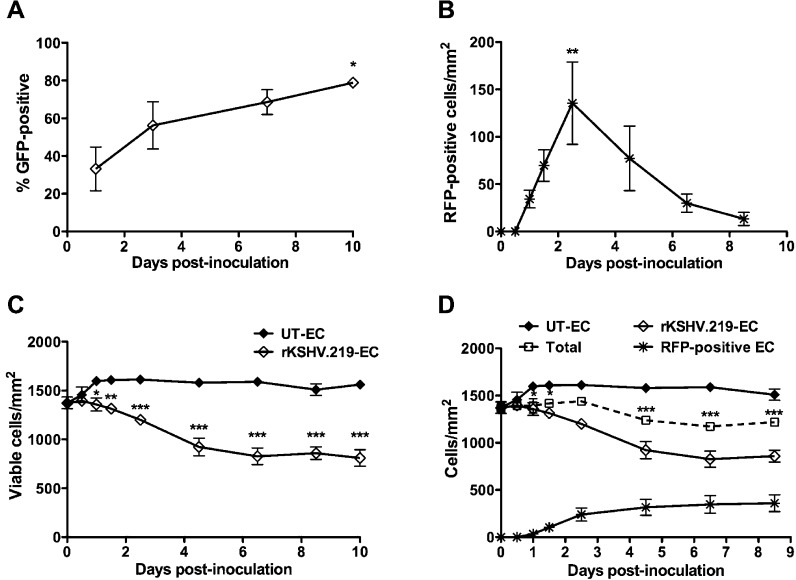
Effect of time on GFP and RFP expression and monolayer density in rKSHV.219 inoculated cultures of endothelial cells. Endothelial cells were untreated (UT-EC) or inoculated with rKSHV.219 (MOI 10) (rKSHV.219-EC) and maintained in culture for analysis of percentage of: (A) GFP-positive cells, (B) RFP-positive cells (*P < 0.05 or **P < 0.01 for comparison to 1 or 0 dpi respectively in Dunnett's multiple comparison posttests following one-way ANOVA) and (C) viable cell density (*P < 0.05, **P < 0.01, or ***P < 0.001 for comparison to UT-EC in Bonferroni posttests following two-way ANOVA). (D) Contribution of lytic death to the lower cell density with rKSHV.219 inoculation showing: total viable cell number in rKSHV.219-EC and UT-EC, number of RFP-positive rKSHV.219-EC (plotted cumulatively), and total (lytic + viable) cell number enumerated for rKSHV.219-EC cultures (*P < 0.05 or ***P < 0.001 compared to UT-EC in Bonferroni posttests following two-way ANOVA). Data are mean ± SEM for 3–4 experiments.

To characterise the temporal development of rKSHV.219 infection within endothelial cell monolayers, phase contrast, GFP and RFP images of untreated and rKSHV.219-inoculated cultures were taken at various time points up to 10 dpi and from these images, the viable and RFP-positive (lytic) cell numbers were determined. The percentage of GFP-positive cells was assessed by flow cytometry. Untreated endothelial cells maintained characteristic cobblestone morphology throughout (Fig. 2). In contrast, by 3 dpi some GFP-positive cells within the rKSHV.219-inoculated cultures had an elongated spindle shape and the number of GFP-positive cells with this altered morphology increased over time (Fig. 2). The percentage of GFP-positive cells increased temporally, from around 30% at 1 dpi to 80% by 10 dpi (Fig. 3A). Conversely, the maximum presence of RFP-positive lytic cells occurred around 2 dpi, and the extent of lytic reactivation subsequently declined to baseline levels (Fig. 3B). Approximately 15% of cells within the rKSHV.219-inoculated cultures died due to lytic death in the first 2.5 days. To investigate if the temporal increase in GFP-positive cells within rKSHV.219-inoculated cultures could be due to the enhanced replication of the rKSHV.219-infected cells compared to the uninfected cells, the cell densities of the untreated and rKSHV.219-inoculated endothelial cell monolayers were counted from the phase contrast images at time points prior to, and up to 10 days after inoculation (Fig. 3C). Densities of untreated monolayers increased up to 24 h post-seeding, before reaching a plateau, whereas rKSHV.219 inoculation caused a progressive decrease in monolayer density from 12 hpi, such that rKSHV.219-inoculated monolayers were significantly different in density compared to those of untreated control cultures by 1 dpi (Fig. 3C). To assess whether the lower monolayer density with inoculation was due to the loss of rKSHV.219-infected cells that underwent lytic replication, the numbers of RFP-positive cells that evolved between time points (Fig. 3B) were summed to give, for each time point, the total number of cells that had undergone lytic replication since the time of inoculation (Fig. 3D). Addition of this profile to the viable cell density (open diamonds, Fig. 3D) for the inoculated cultures gave the theoretical total monolayer density for these cultures (open squares, Fig. 3D). Since total monolayer density did not equal the density of the untreated control at 4.5 dpi and beyond, the lower densities of the inoculated cultures could not be completely explained by lytic death. However, the total densities of the rKSHV.219-inoculated cultures at 0 dpi and 8.5 dpi were not significantly different (*P* < 0.05 by paired *t*-test). Thus there was no loss of cell number within the inoculated monolayers that was not explained by lytic death. Therefore, the higher numbers of viable cells in the uninfected monolayers were most probably a consequence of inhibition of proliferation in monolayers inoculated with rKSHV.219. Since the cell number associated with rKSHV.219-inoculted cultures did not increase over time, even as lytic reactivation subsided, it was concluded that rKSHV.219-inoculation did not induce endothelial cell proliferation. Therefore, proliferation did not account for the increase in GFP-positive cells in rKSHV.219-inoculated cultures over time.

### 3.3 Characterisation of rKSHV.219 transmission through endothelial cell cultures

**Fig. 4:**
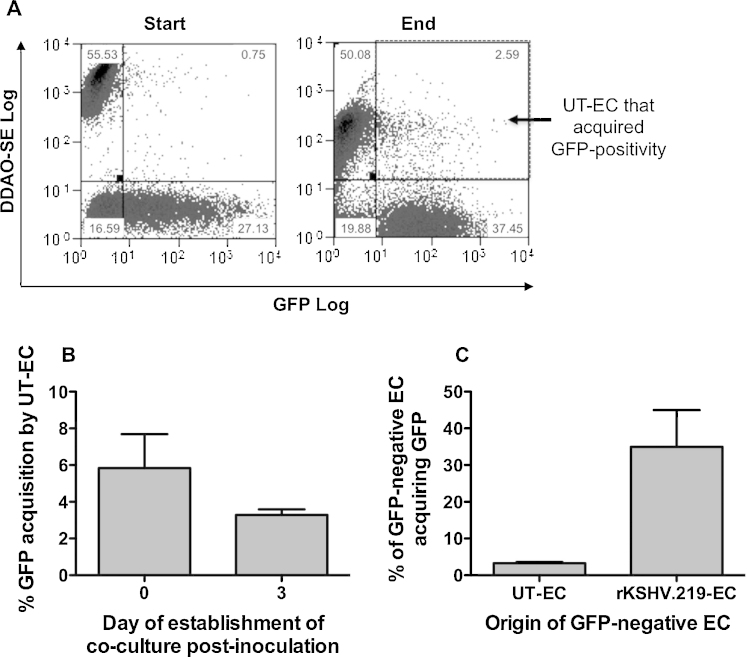
Quantification of horizontal transmission of rKSHV.219 infection through inoculated endothelial cell cultures. Endothelial cells were inoculated with rKSHV.219 (MOI 10) and maintained in culture for 0 or 3 days before they were mixed with DDAO-SE-labelled untreated endothelial cells (UT-EC) and co-cultured for 5 days. Proportions of DDAO-SE positive UT-EC or GFP-negative, DDAO-SE negative cells that acquired GFP-positivity by the end of the 5d culture period were assessed to detemine rKSHV.219 re-infection. (A) GFP Log v DDAO-SE Log plots for a representative assay fixed at the start and end of the 5 day co-culture period. In the assay, single population controls including: non-labelled UT-EC; DDAO-SE-labelled UT-EC; and non-labelled rKSHV.219-EC, as well as a co-culture control of non-labelled UT-EC and DDAO-SE-labelled UT-EC, were established in parallel to verify that double positive cells were not due to dye transfer or to the positioning of the quadrants for analysis. (B and C) Quantification of the data illustrated in (A) showing: (B) the percentage of DDAO-SE positive UT-EC that became GFP-positive during the 5 day co-culture for cultures established immediately after inoculation (0 days) and 3 dpi; (C) the percentage transmission of infection to uninfected cell populations within the co-culture (either DDAO-SE positive UT-EC or DDAO-SE negative rKSHV.219-EC) during the 5 day co-culture, for cultures established 3 dpi. Data are mean ± SEM for 2–3 experiments.

Next, this study sought to determine if the temporal increase in GFP-positive cells in rKSHV.219-inoculated endothelial cell cultures was due to horizontal transmission of virus to previously uninfected cells. To address this question, untreated cells, labelled with DDAO-SE were co-cultured with non-labelled, rKSHV.219-inoculated cells from the same donor. This co-culture was for 5 days and was established either immediately following inoculation (0 dpi, following removal of free virus) or 3 dpi. Results were analysed using flow cytometry at the beginning and end of the co-culture period (Fig. 4A), allowing determination of the acquisition of GFP-positivity by untreated DDAO-SE positive cells co-cultured with rKSHV.219-inoculated endothelial cells (Fig. 4A). The DDAO-SE staining faded during the period of co-culture but remained adequate in intensity for labelled cells to be distinguished from non-labelled cells. There was low, but consistently detectable, transmission of GFP to untreated DDAO-SE positive non-inoculated endothelial cells, both when co-cultures were established immediately following inoculation and, to a lesser extent, when co-cultures were established at 3 dpi (Fig. 4B). This was consistent with earlier data, which showed that the number of RFP-positive cells peaked early after inoculation (Fig. 3B); therefore, presumably there was more rKSHV.219 being produced and capable of re-infection in the co-cultures established early after infection. However, horizontal transmission did not account for the increase in GFP-positivity seen in the rKSHV.219-inoculated cultures over time (Fig. 3A), as 35% of GFP-negative DDAO-SE negative cells from the original rKSHV.219-inoculated cultures acquired GFP expression between 3 and 8 dpi, in contrast to only 3.3% of DDAO-SE positive non-inoculated endothelial cells by horizontal transmission (Fig. 4C).

### 3.4 Analysis of the temporal relationship between rKSHV.219 GFP and LANA-1-expression

**Fig. 5:**
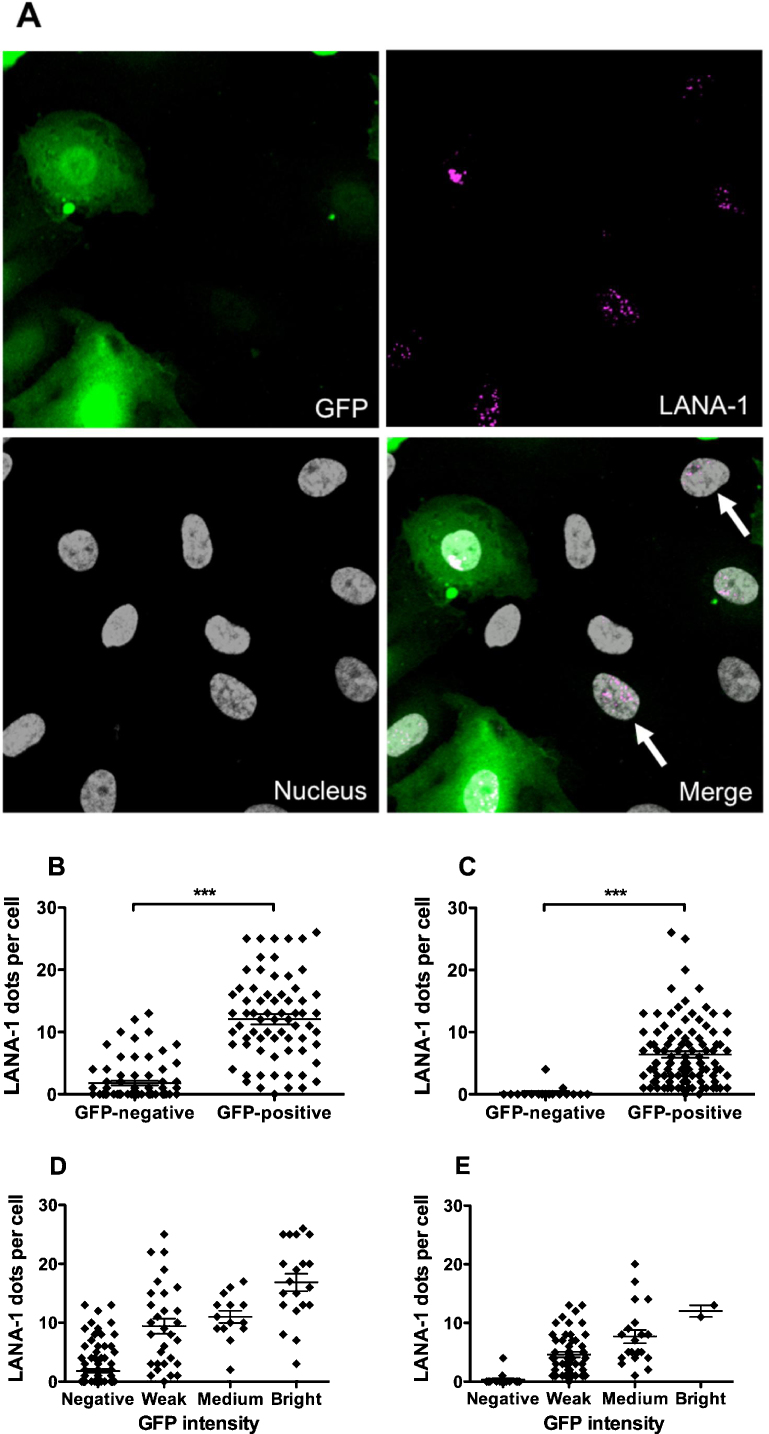
Comparison of LANA-1 and GFP as markers of rKSHV.219 infection of endothelial cells. Endothelial cells were inoculated with rKSHV.219 (MOI 10) and maintained in culture prior to staining for, and quantification of, the number of LANA-1 dots in GFP-negative or GFP-positive cells at (B) 24hpi, or (C) 10dpi (***P<0.001 by unpaired t-test). (A) Representative image of a KSHV-inoculated endothelial cell culture stained for LANA-1 at 24h. Arrows indicate GFP-negative, LANA-1 positive cells. GFP Frequency of LANA-1 dots versus GFP intensity at (C) 24hpi or (D) 10dpi. ANOVA showed a significant effect of GFP intensity on LANA-1 dot frequency (P<0.0001).

As the increase in percentage GFP-positive cells within rKSHV.219-inoculated cultures over time could not be explained by an increased proliferative capability of infected cells, or transmission of infection between cells, this study went on to investigate the possibility that the total number of GFP-positive cells seen at 10 dpi was established during initial inoculation, and the time required for intracellular amplification of the GFP signal varied between individual cells. rKSHV.219-inoculated cultures were stained, at either 24 h or 10 dpi, with an antibody against KSHV latency-associated antigen-1 (LANA-1), as an alternative confirmation of infection. LANA-1 stained with a distinct punctate nuclear pattern as previously described (Kedes et al., 1997; Fig. 5A). Previous studies have shown the number of individual LANA-1 dots to be directly proportional to the amount of intracellular viral DNA (Adang et al., 2006). At 24 hpi all GFP-positive cells stained positive for one or more LANA-1 dots (Fig. 5A). Interestingly, however, LANA-1 dots were also detected in 37% of GFP-negative cells within the rKSHV219-incolculated culture at that time point (Fig. 5A and B), although the number of dots per cell was significantly lower than that seen in the GFP-positive group. At 10 dpi 98% of GFP-positive cells were LANA-1 positive (Fig. 5C), and only 12% of GFP-negative cells were LANA-1 positive. These data suggested that the temporal development of GFP-positivity was related to initial viral load. To investigate this idea further, cells were grouped according to their GFP fluorescence intensity into the categories negative, weak, medium or bright. An association between the intensity of GFP expression and the number of LANA-1 dots was evident at both 24 hpi and 10 dpi (Fig. 5D and E). Interestingly, the overall number of LANA-1 dots in each infected cell was significantly lower at 10 dpi compared to 24 hpi (*P* < 0.0001 by unpaired *t*-test). Cells were also less bright at 10 dpi than 24 h, possibly this indicated a loss of viral episomes over time. Overall these data support further the hypothesis that time for amplification of viral copy number and/or GFP accumulation to detectable levels in cells that receive a lower viral load accounts for the delay in GFP-positivity observed in this system. Taken together, Fig. 5D and E indicates that viral gene expression, yielding 5–10 LANA-1 dots, is the threshold for GFP-positivity in this system.

## 4 Discussion

The development of a recombinant KSHV (rKSHV.219) that has been engineered to express GFP or RFP, indicating latent or lytic infection respectively, has provided an extremely useful tool to the KSHV research community. This study provides a detailed description of the infection and transmission dynamics of this virus in primary endothelial cells and discusses the validity of GFP as a marker of infection of these cells using primary umbilical vein endothelial cells as the model endothelial cell type. Initial rKSHV.219 MOI and monolayer density modulated the resultant percentage of GFP-positive cells. The percentage of GFP-positive cells in rKSHV.219-inoculated cultures increased over time, whilst the number of RFP-positive cells peaked at 2 dpi. This lytic burst did not lead to substantial transmission of newly produced virus to uninfected cells. Indeed, the temporal increase in the percentage of GFP-positive cells was related to the number of rKSHV.219 copies acquired per cell during initial inoculation. This study also identifies that GFP is not a reliable indicator of rKSHV.219 infection of endothelial cells at early time points post-inoculation, as GFP-negative cells within rKSHV.219-inoculated cultures were frequently LANA-1-positive. However, by 10 dpi there was a greater concordance between GFP and LANA-1 expression, revealing that GFP was a reliable indicator for rKSHV.219 infection at later time points. Overall, this report not only provides useful and important information for researchers using rKSHV.219 to infect primary endothelial cells, but also raises important factors to consider when conducting research with viruses engineered to express fluorescent proteins from cellular promoters as a marker of infection.

The temporal increase in the percentage of GFP-positive cells within rKSHV.219-inoculated cultures, which this study identified, was consistent with previous reports on KSHV infection of endothelial cells (Butler et al., 2011, Moses et al., 1999). Others suggested that such an increase could be a consequence of an enhanced proliferation of infected cells, or horizontal transmission of KSHV virions (Moses et al., 1999). However, in this system, rKSHV.219 did not increase endothelial cell proliferation; indeed, cell numbers were consistently lower in the rKSHV.219-inoculated cultures than in the untreated control cultures, a phenomenon that was mostly explained by the death of infected cells from lytic replication. Secondly, the occurrence of horizontal transmission of rKSHV.219 from infected to untreated cells was minimal, and significantly (indeed, over 10 times) lower than the increase in GFP-positive endothelial cells arising in rKSHV.219-inoculated cultures over time. Thus it appeared that in this system, the increase in the percentage of GFP-positive cells over time was a consequence of non-uniform loading of the endothelial cells with rKSHV.219 virions during the initial inoculation, meaning that where fewer viral copies were originally delivered to the cells, there was a longer delay before detectable levels of GFP could be identified using the methods stated. Previous studies have reported that de novo infection with KSHV can result in highly varied levels of intracellular KSHV load, which correlate with both the total LANA-1 fluorescence intensity and the number of LANA-1 dots (Adang et al., 2006). Why LANA-1 expression is higher in some cells than others has not been described, but one speculation is that it could be due to variation between individual cells within the culture. For example, the cell cycle status of a cell could affect its propensity for infection, as has been described for papillomavirus (Pyeon et al., 2009). Previous work has shown that endothelial cells isolated from umbilical cords contain a mixed population, with both mature and progenitor endothelial cells in the resultant culture (Ingram et al., 2004); therefore intra-culture variation in the expression of molecules required for KSHV entry or LANA-1 expression might also be involved. In this study, LANA-1 was found expressed at low levels in almost 40% of GFP-negative cells at 24hr following rKSHV.219-inoculation. Those cells that had a higher viral load (i.e. a higher frequency of LANA-1 dots) were more likely to be GFP-positive at this early time point. At 10 dpi, GFP expression correlated more closely with the presence of LANA-1, although interestingly, the average number of LANA-1 dots per cell was lower at this later time point, suggesting loss of episomes with culture duration, consistent with previous reports in other cell types (Grundhoff and Ganem, 2004). The intensity of GFP signal at this later time point may not be an accurate measure of episome number itself, due to the dynamics of episome loss vs. GFP loss over time. Thus, for a conclusive assessment of the presence and quantity of rKSHV.219 in each cell it would be necessary to stain for a viral protein, such as LANA-1. In addition, qRT-PCR could help to provide a more definitive measurement of episome number and absolute viral load in the culture.

Whether it is important to consider the LANA-1 positive, GFP negative, cells within the culture when studying the functional consequences of infection is so far unknown. Qualitative assessments of cell shape and GFP expression at 24 hpi in this study concluded that cells with the strongest GFP expression underwent the greatest morphological change. Furthermore, previous work showed that the reduction of ICAM-1 expression on rKSHV.219-inoculated endothelial cells was more pronounced for the highly GFP-positive cells, compared to those that expressed less GFP (Butler et al., 2011). Thus, it is likely that varying thresholds of virion number are required to modify KSHV-induced cellular processes within a defined time frame. However, it might be assumed that the presence of even a single virion in the cell could modify its phenotype when compared to an uninfected cell; therefore GFP-negative, LANA-1-positive cells cannot be assumed equivalent to untreated cells, and further studies are required to establish the relationships linking GFP expression, number of LANA-1 dots and function.

In conclusion, this study provides a useful characterisation of rKSHV.219 infection of primary endothelial cells. In addition, it raises important points to be considered when using GFP expression as a definitive marker of rKSHV.219 infection.
