# Supplementary material for: Infection and transmission dynamics of rKSHV.219 in primary endothelial cells
Source: J Virol Methods. 2013 Oct;193(1):251–9. doi: 10.1016/j.jviromet.2013.06.001 (PMC4147964; doi:10.1016/j.jviromet.2013.06.001)
Supplement: Supplementary file 1 [file mmc1.pdf]

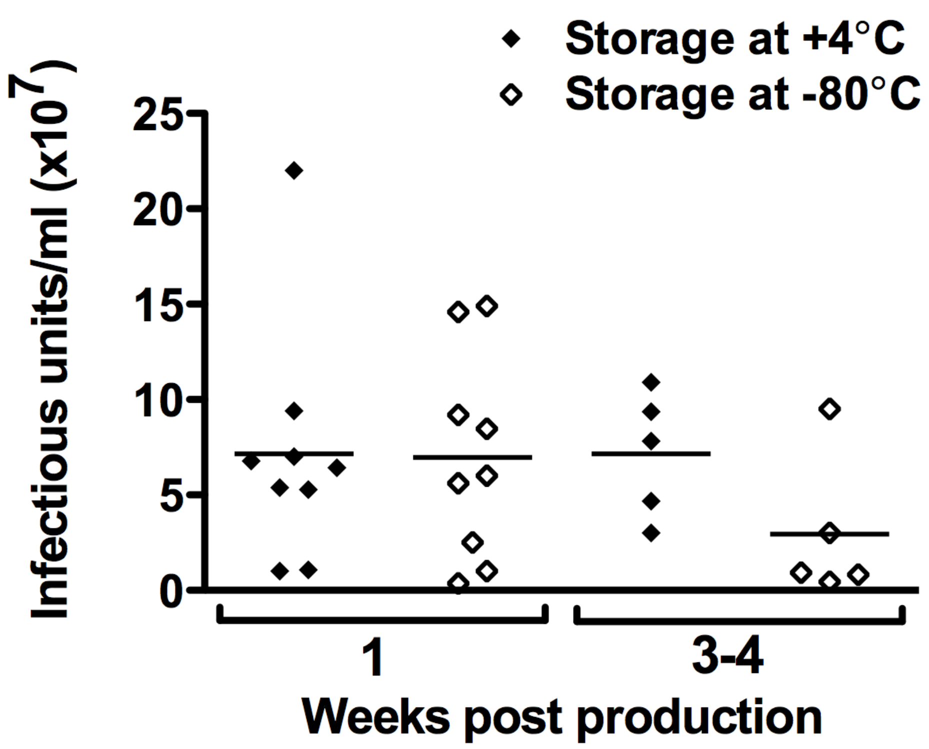

Supplementary Figure 1: Effect of storage temperature upon rKSHV.219 titre. rKSHV.219 preparations were aliquoted immediately following production and stored at 4□C or -80□C. Titres were assessed using HEK293 cells after 1 or 3-4 weeks storage. Each point represents the test results for a separate preparation.

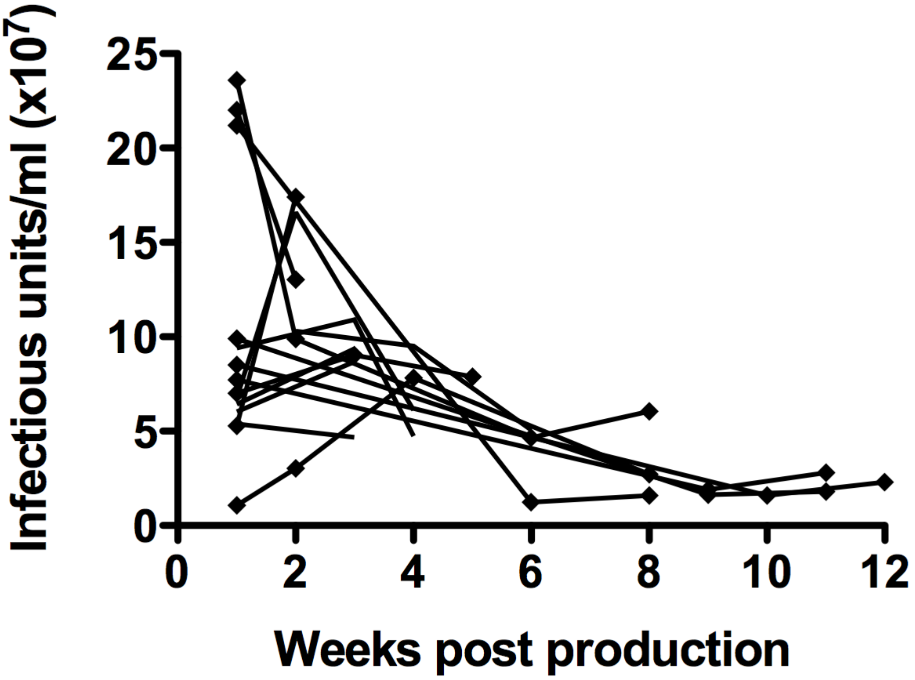

Supplementary Figure 2: Effect of storage time upon rKSHV.219 titre.

The titres of rKSHV.219 preparations stored at 4°C were analysed at time points up to 12 weeks post-production by titration on HEK293 cells. Each line represents a different virus preparation.
